# Battle of the sexes: Understanding donor:recipient sex differences in transplantation biology

**DOI:** 10.1002/hem3.70000

**Published:** 2024-09-10

**Authors:** David G. Kent

**Affiliations:** ^1^ Department of Biology, Centre for Blood Research, York Biomedical Research Institute University of York York UK

Studying hematopoietic stem cell (HSC) function is most powerfully done with serial transplantation assays that can formally demonstrate the hallmark functional properties of durability, self‐renewal, and multilineage differentiation capacity. Transplantation assays have taken many forms over the decades to provide evidence of HSC function, with mouse:mouse studies representing the bulk of studies to date. Competing for the limelight as a “gold standard” assay for nearly as long, however, is the human HSC:mouse recipient xenotransplantation assay, which has been powerfully used to help define the relative function of both normal HSCs and HSCs isolated from leukemia patients. The latter presents the most urgent need for establishing robust assays in the hematological community. It represents the opportunity to study the function of HSCs isolated from patients with the diseases that we are trying to cure. While there are a number of studies that have used human:human transplantations in clinical settings to study the dynamics of HSCs,^1^, ^2^ the ability to characterize leukemic HSCs at a detailed molecular level and to treat them with experimental compounds to potentially modify those clonal dynamics for therapeutic purposes remains extremely limited. This is where the xenotransplantation assay comes to the fore, but defining an agreed set of standards in the field is a complex business, and a recent study in *HemaSphere* by Mian et al. sheds some light on one of the key factors in transplantation biology—sex differences.^3^

Sex differences in transplantation have been known about for some time, although the studies do not always agree on the how's and why's of these differences. Single HSC transplantation studies in mouse:mouse donor:recipient pairs showed that male HSCs did not perform well in female recipients, potentially due to a weak antigen coded for by the Y chromosome.^4^ Another study,^5^ in human:mouse xenotransplants, showed that female immunodeficient NOD/SCID/IL‐2Rgc‐null (NSG) mice were far superior as recipients of human cells with increases in both engraftment and proliferation of human HSCs (and this was also evidenced at the single‐cell level). In this latter study, two potential reasons were speculated: first, that “female NSG mice might be more immunodeficient than males,” and second that “sex‐associated factors, such as steroid hormones, can positively or negatively regulate human HSCs.” It is clear that sex matters, but with the emergence of new immunodeficient models and a general lack of comparative studies between male and female recipients, it is difficult to articulate a set of field recommendations for how to undertake xenotransplantation experiments with precious patient samples.

The recently published study by Mian et al.^3^ goes some way to addressing this. Using various immunodeficient mouse models, they studied engraftment levels and mature cell production across a range of normal and malignant samples and observed substantial sex differences between these models and blood cell outputs. First, they established that NSG‐3/GM/SF (NSG‐SGM3) mice are superior compared to NSG and NSG with a ckitW41 mutation (NBSGW) mice for the engraftment of acute myeloid leukemia (AML) patient samples (50% in NSGS vs. <25% for either NSG or NBSGW). Importantly, they also showed that AML cells, unlike normal HSCs, which can exhaust in NSGS mice,^6^ performed well in secondary transplantations, potentially due to the increased self‐renewal properties of leukemic HSCs.

Where the data become really interesting, however, is when the sex of the human donor and mouse recipient cells are considered. Here, primary AML cells were shown to have sex‐specific engraftment potential that was dependent on the sex of the donor and recipient cells. They first found that female patient samples consistently generated significantly lower levels of human cell output (>3‐fold difference on average). Next, they showed that transplanting female patient cells into male mouse recipients gave little to no engraftment, whereas in female mouse recipients, levels were much higher. This difference in recipient specificity was not observed for male patient samples, clearly pointing to the importance of considering the sex of both donor and recipient cell type in these types of experiments. Notably, the differences in recipient sex were mirrored in both the NSG and NSGS recipients. Following these retrospective observations, they next undertook a set of experiments to directly test the hypothesis that female AML cells would perform better in female recipients and in all three female AML cells (and none of the four male AML samples); engraftment levels were improved upon transplantation into female recipients. The authors finish the paper off with comparisons of mature cell outputs and quiescence status in normal HSCs transplanted into male and female recipients and note interesting patterns of mature cell production that also depend on the sex of the donor and recipient.

Together, these findings highlight the importance of donor:recipient sex‐matching in transplantation studies and also highlight the need for reporting recipient sex in research reports. It further suggests that sex‐specific mechanisms should be explored further in the context of transplantation biology and clinical trial design, ensuring that if sex‐specific differences in the biology of HSCs and their progeny exist, then they should be accounted for. In the coming years, it will be very interesting to see how such observations move forward in the design and reporting of experiments.

## AUTHOR CONTRIBUTIONS

D. G. K. is the sole contributor to this article.

## CONFLICT OF INTEREST STATEMENT

The author declares no conflicts of interest.

### FUNDING

Work in the D. G. K. laboratory is supported by the Bill and Melinda Gates Foundation (INV002189), an ERC Starting Grant (ERC‐2016‐STG–715371), a Cancer Research UK Programme Foundation Award (DCRPGF\100008), and the Medical Research Council (MC_PC_21043).

## Data Availability

Data sharing is not applicable to this article as no new data were created or analyzed in this study.
